# Observations on the Proximate Cause of Insanity

**Published:** 1844-04

**Authors:** 


Art. IV. — *Observations on the Proximate Cause of Insanity.* By James Sheppard, m.r.c.s., *being an attempt to prove that insanity is dependent on a Morbid Condition of the Blood.* — *London,* 1844. 12mo.

One of our most irksome duties as reviewers is to read such a book as Mr. Sheppard's; which, under other circumstances, we should throw aside as useless after the perusal of a very few pages. Mr. Sheppard tells us that whilst reflecting on a drunken man he was impressed with the idea that insanity was at times connected with morbid conditions of the blood,—a very probable supposition; but on further reflection he came to the conclusion that there is presumptive evidence "that insanity is *universally* and essentially dependent on morbid conditions of the blood." This he "attempts to prove" by the faulty and exploded process of first framing a crude and hasty generalization from a few facts or opinions, and then finding or bending facts and arguments to prove it. He has brought forward no new facts, nor is there any internal evidence that the writer is at all practically acquainted with insanity. He has collected a few well-known truths showing that delirium and mania may coexist with excess in the quantity of the blood, and is relieved by depletion, and that puerperal mania is often the result of a deficiency in the quantity of the blood; but on the most important point, the alteration of its quality in insanity, we have none of that evidence which alone would be at all conclusive. By chemical analysis of the blood of the insane some new and useful information might have been collected, and in a book with this title such information was to be expected; but instead of cautious inferences drawn from pains-taking chemical research, in connexion with a personal acquaintance with the disease, we find that the author has been seeking fame by a much easier process, by collecting from a few well-known books the opinions of those authors most favorable to his own view, drawing from their opinions the loosest conclusions, and when these fail, putting "I doubt not . . I firmly believe . . I apprehend . . I can easily imagine" as the grounds on which we are also not to doubt, but to imagine easily, or believe firmly.

To show that we are not unfairly severe, we will quote three conclusions to which the author arrived:

"I have, I hope, made it appear in the preceding pages—

"1st. That insanity does not depend on disease of the brain.

"2d. That insanity cannot result from unappreciable lesion of structure.

"3d. That no conclusive evidence can be adduced in support of the position, that insanity can result from morbid conditions of the nervous system." (p. 33-4.)

And how have these conclusions been arrived at on these three important questions? By the numerical method? By a collection of original cases carefully analysed? By personal acquaintance with the disease during life, and by examinations after death? Or even by a deep acquaintance with the mere literature of the malady? By no means. But by a few well-known cases from a work of Dr. Abercromby's, and a few quotations of the *opinions* of a few writers on insanity, with the old experiment of Mr. Morgan's on the action of poisons, all made the basis of loose, inconclusive reasoning. Need we say that by this method truth can never be arrived at, or the bounds of medical science enlarged? The condition of the blood in so obscure a disease as insanity is well worth investigating, and it should be subjected to chemical analysis by those who have the competent skill, the results compared with the analysis of blood in other diseases, and the connexion between its condition and the symptoms traced by those who are practically acquainted with the complaint. By some such process as this the question as to the relation between the blood and insanity may be settled, not by hastily assuming the fact that there is such a relation, and then proving it by words. It is true that the former method requires great opportunity of investigating the disease, chemical knowledge and skill, a cool judgment, and that rare quality the power of original observation; and that the latter is very easy, only needing a few books, pen, ink, paper, a lively fancy, no humility, and a very considerable opinion of one's own powers: but the one may benefit mankind by throwing light on one of the darkest defects of humanity; the other merely indulges an individual's egotism, vanity, and self-conceit.

